# Genome-Wide Control of RNA Polymerase II Activity by Cohesin

**DOI:** 10.1371/journal.pgen.1003382

**Published:** 2013-03-21

**Authors:** Cheri A. Schaaf, Hojoong Kwak, Amanda Koenig, Ziva Misulovin, David W. Gohara, Audrey Watson, Yanjiao Zhou, John T. Lis, Dale Dorsett

**Affiliations:** 1 Edward A. Doisy Department of Biochemistry and Molecular Biology, Saint Louis University School of Medicine, Saint Louis, Missouri, United States of America; 2 Department of Molecular Biology and Genetics, Cornell University, Ithaca, New York, United States of America; 3 The Genome Center, Department of Genetics, Washington University in St. Louis School of Medicine, Saint Louis, Missouri, United States of America; Ludwig Institute for Cancer Research, University of California San Diego, United States of America

## Abstract

Cohesin is a well-known mediator of sister chromatid cohesion, but it also influences gene expression and development. These non-canonical roles of cohesin are not well understood, but are vital: gene expression and development are altered by modest changes in cohesin function that do not disrupt chromatid cohesion. To clarify cohesin's roles in transcription, we measured how cohesin controls RNA polymerase II (Pol II) activity by genome-wide chromatin immunoprecipitation and precision global run-on sequencing. On average, cohesin-binding genes have more transcriptionally active Pol II and promoter-proximal Pol II pausing than non-binding genes, and are more efficient, producing higher steady state levels of mRNA per transcribing Pol II complex. Cohesin depletion frequently decreases gene body transcription but increases pausing at cohesin-binding genes, indicating that cohesin often facilitates transition of paused Pol II to elongation. In many cases, this likely reflects a role for cohesin in transcriptional enhancer function. Strikingly, more than 95% of predicted extragenic enhancers bind cohesin, and cohesin depletion can reduce their association with Pol II, indicating that cohesin facilitates enhancer-promoter contact. Cohesin depletion decreases the levels of transcriptionally engaged Pol II at the promoters of most genes that don't bind cohesin, suggesting that cohesin controls expression of one or more broadly acting general transcription factors. The multiple transcriptional roles of cohesin revealed by these studies likely underlie the growth and developmental deficits caused by minor changes in cohesin activity.

## Author Summary

The cohesin protein complex binds to chromosomes and helps ensure that chromosomes are divided equally into the daughter cells when a cell divides. Cohesin also affects how genes are expressed. Small changes in cohesin alter gene expression and development, causing Cornelia de Lange syndrome, a genetic disease. Cohesin influences the amount of RNA produced by many genes, but the reasons are poorly understood. We investigated this question by measuring how changes in cohesin levels affect the level of RNA polymerase, the enzyme that transcribes genes to make RNA, at all genes in *Drosophila* cells. We find that genes that bind cohesin have higher average levels of RNA polymerase and produce more final processed RNA per RNA polymerase than genes that don't bind cohesin. We also find that cohesin binds nearly all DNA sequences located outside of genes that are predicted to regulate gene expression. Reducing cohesin affects RNA polymerase levels at many genes and the predicted regulatory sequences, indicating that cohesin facilitates communication between regulatory sequences and genes. Our data also show that cohesin affects transcription of most genes that don't bind cohesin, likely by controlling transcription of broadly acting transcription factors that regulate many genes.

## Introduction

Cohesin is a large protein ring that topologically encircles DNA and participates in several chromosome functions, including sister chromatid cohesion, chromosome segregation, DNA repair, and gene expression (reviewed in [1]–[3]). It is loaded onto chromosomes by the kollerin complex, and removed by the releasin complex.

Modest changes in cohesin, kollerin or releasin activity alter gene expression, growth, and animal development without measurable defects in chromatid cohesion or chromosome segregation. For instance, minor alterations of kollerin or cohesin activity in humans cause Cornelia de Lange syndrome (CdLS, OMIM #122470, #300590, #610759, #614701) which is associated with significant physical and intellectual deficits (reviewed in [4]). Cohesin also influences gene expression in non-dividing cells [5], [6]. Thus, cohesin's role in gene expression appears largely independent of its role in cell division, and considerably more sensitive than its other cellular functions to changes in cohesin dosage.

Current evidence argues that cohesin directly influences gene transcription. In animal cells, cohesin and kollerin preferentially bind genes important for growth and development near the transcription start site and in the transcribed region [7]–[11]. In Drosophila, cohesin is largely absent from silent genes, and selectively binds active genes in which transcriptionally-engaged RNA polymerase II (Pol II) pauses just downstream of the start site [9], [12]. Upon depletion of cohesin or kollerin, mRNAs from cohesin-binding genes are more likely to be affected than those from non-binding genes, and can change within a few hours [5], [13]. Current evidence argues that cohesin regulates transcription by multiple mechanisms, including facilitating enhancer-promoter and insulator looping, and by controlling the transition of promoter-proximal paused Pol II to efficient elongation [1], [2].

The prior studies of how cohesin regulates gene expression measured steady state mRNA levels, and thus do not clearly differentiate the roles of cohesin in transcription from other processes such as RNA splicing, transport, and stability. To gain more direct insights into the mechanisms by which cohesin influences transcription, we measured the effects of cohesin depletion on the genome-wide distribution of Pol II, Pol II phosphorylated at the serine 2 residue in the heptad repeats in the C terminal domain of the Rpb1 subunit (Ser2P Pol II), P-TEFb, and Cdk12 in Drosophila cells derived from central nervous system. Ser2P Pol II is actively elongating and formed by the action of the P-TEFb and Cdk12 kinases. We also measured the effects of cohesin and kollerin depletion on transcriptionally-engaged Pol II by precision global run-on sequencing (PRO-seq). We deduce that cohesin directly promotes the transition of promoter-proximal paused Pol II to elongation at many genes that it binds from comparing the changes in Pol II occupancy and activity in control and cohesin-depleted cells. The evidence indicates, that in many cases, cohesin likely facilitates this transition by supporting long-range enhancer-promoter interactions, but also has other roles directly at the promoter. Surprisingly, we also find that cohesin influences Pol II activity at most genes that don't bind cohesin, possibly through control of broadly-acting transcription factors.

## Results

### Cohesin preferentially binds genes with higher levels of Pol II and promoter-proximal transcriptional pausing

To directly assess the influence of cohesin on gene transcription, we compared the genome-wide occupancy of Pol II and Pol II kinases relative to cohesin binding, and measured the effects of cohesin depletion on Pol II and kinase occupancy. We used genome-wide chromatin immunoprecipitation with tiling microarrays (ChIP-chip) to measure the genome-wide binding of Pol II, the Cyclin T (CycT) subunit of the P-TEFb complex, and the Cdk12 Pol II kinase in ML-DmBG3 (BG3) Drosophila cells derived from larval central nervous system. We used antibodies against the Rpb3 subunit of Pol II [14] to measure the total Pol II occupancy, and antibodies specific for Ser2P Pol II to measure elongating Pol II. All ChIP-chip experiments were performed with two independent biological replicates and averaged.

**Figure 1:**
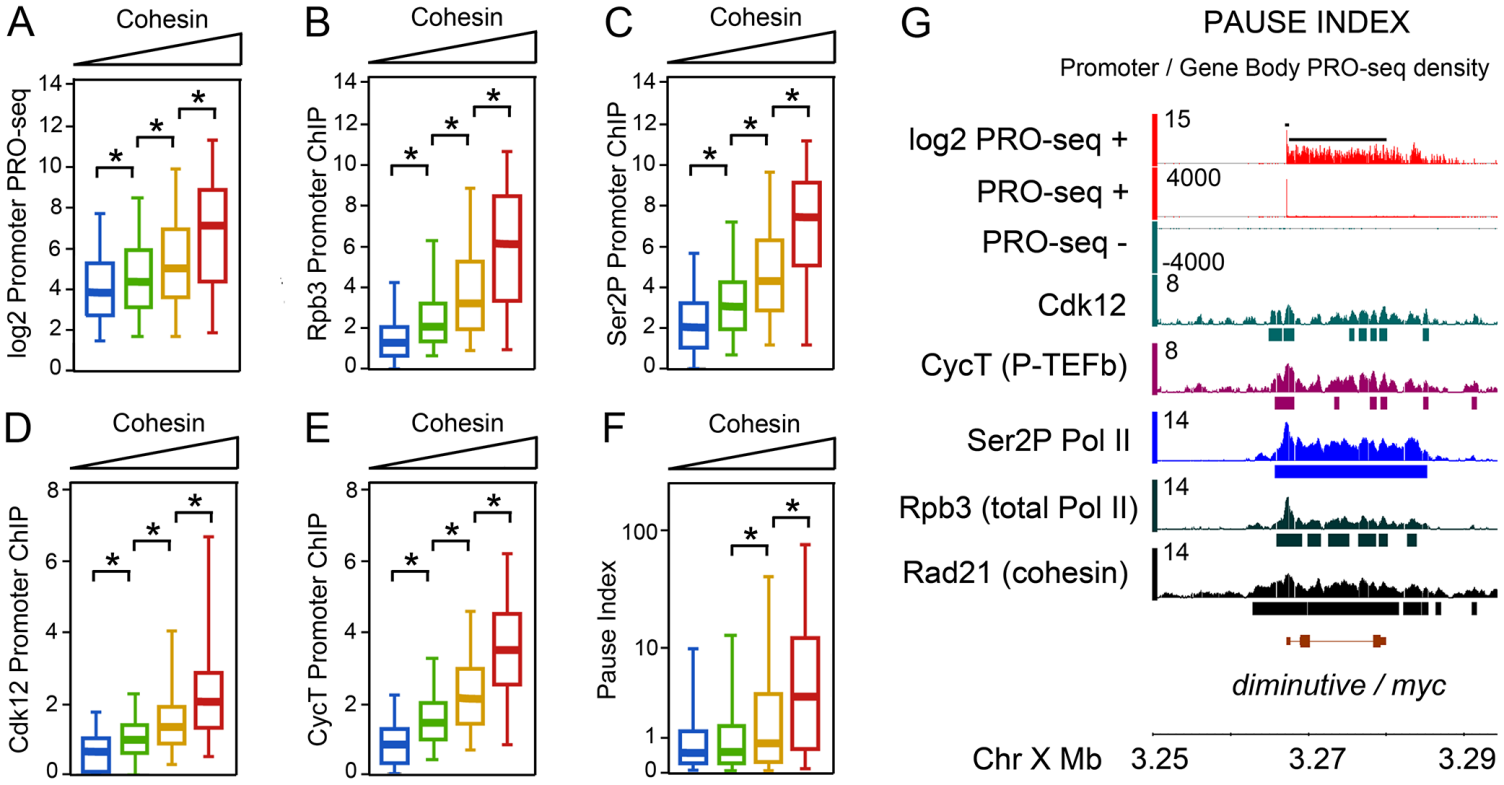
Pol II occupancy and promoter-proximal pausing at active genes correlates with cohesin levels in BG3 cells. (A) PRO-seq density at the promoters of active genes divided into four groups with increasing levels of cohesin at the promoter as measured by Rad21 ChIP-chip. Active genes have at least 1 read per million in both the promoter and gene body regions. The four cohesin groups and number of genes in each are shown in Figure S1A. The box plots show the distributions of promoter PRO-seq density for each cohesin group. Asterisks indicate significant differences as determined by Kolmogorov–Smirnov statistical tests. (B–E) Rpb3, Ser2P Pol II, Cdk12, and CycT occupancy of active genes as measured by ChIP-chip. (F) Pause index distributions for each cohesin level group. Pause index was calculated using PRO-seq data (see text). (G) Example of PRO-seq and ChIP-chip data at the *diminutive* (*myc*) gene. The black bars above the log2 PRO-seq track indicate the regions defined as promoter (200 bp surrounding the transcription start site) and gene body (rest of annotated transcription unit). Bars beneath the ChIP-chip tracks indicate where occupancy is significant at p≤10^−3^.

Genome-wide, Rpb3 correlates well with Ser2P Pol II (r = 0.87), especially on gene bodies (Table 1; Figure 1). Pol II positively correlates with the CycT subunit of the P-TEFb Pol II kinase (0.64–0.67), and somewhat less, although significantly, with the Cdk12 kinase (0.39–0.45) (Table 1). The Rad21 cohesin subunit strongly overlaps Pol II (r = 0.67–0.68), consistent with prior findings [9], and has a similar correlation with CycT (0.73), but less with Cdk12 (0.49) (Table 1). We often detect CycT and Cdk12 at promoters, and enrichment in the gene bodies is frequently similar in strength, as in *diminutive*, the Drosophila *myc* gene (*dm*, FlyBase FBgn0262656; Figure 1).

**Table 1:** Genome-wide correlations between Pol II, CycT, Cdk12, and Rad21 chromosome association by ChIP–chip.

	Rpb3	Ser2P Pol II	CycT	Cdk12
**Rad21**	0.67	0.68	0.73	0.49
**Rpb3**		0.87	0.67	0.45
**Ser2P Pol II**			0.64	0.39
**CycT**				0.70

ChIP does not determine if Pol II is transcriptionally engaged, or the direction it is transcribing. We thus used precision global run-on sequencing (PRO-seq; [15]), a variation of GRO-seq [16] that gives improved resolution to measure the levels and orientation of transcription-competent Pol II genome-wide. PRO-seq varies from GRO-seq in that biotin-labeled ribonucleotides are used to allow run-on for a nucleotide or two, instead of the longer run-on with BrUTP used in GRO-seq. PRO-seq, like GRO-seq [17], is highly sensitive, and unlike ChIP, does not depend on crosslinking efficiency or antibody specificity, and detects elongation-competent Pol II regardless of the phosphorylation status. Nuclei were isolated under conditions of ribonucleotide depletion to halt transcription, but leave Pol II transcriptionally engaged. The nascent RNA transcripts produced upon restart of transcription were used to generate a cDNA library for high-throughput sequencing. Inclusion of sarkosyl in the run-on transcription reaction prevents new transcription initiation, so that only Pol II that is already transcriptionally engaged is detected, and gene body and promoter paused Pol II are detected with equal efficiency [17]. Two independent biological replicates were used for each PRO-seq measurement (control, Rad21 RNAi, Nipped-B RNAi).

The number of PRO-seq reads was quantified for nearly 17,000 annotated transcription units, and after normalization for the total number of reads, the genome-wide correlations between the two biological replicates were 0.98 for all three groups (Table S1). We selected approximately 7,000 “PRO-seq active” transcription units for detailed analysis by using only those transcription units that had at least 1 read per million in the 200 bp region surrounding the annotated transcription start site, and in the gene body in the control cells (Table S2). Because genes only bind cohesin when they are active [9], restricting the analysis to active genes is essential for valid comparisons of cohesin-binding to non-binding genes. Many genes have more than one active transcription start site, and thus the 7,000 active transcription units represent approximately 6,000 genes.

Cohesin-binding genes have more Pol II on average than non-binding active genes as measured by both PRO-seq and ChIP-chip. When active genes are subdivided into four groups (Figure S1A) from low to high cohesin binding levels based on the mean Rad21 ChIP signal in the 400 bp region surrounding the transcription start site, the average PRO-seq read density and Rpb3, Ser2P Pol II, Cdk12, and CycT ChIP signals at the promoter all increase with cohesin (Figure 1A–1E). Similar results are obtained for both promoters and gene bodies when PRO-seq active genes are split into cohesin-binding and non-binding genes, and Pol II occupancy is measured by ChIP-chip (Figure S1F).

A prior report indicated that cohesin preferentially binds genes with promoter-proximal paused polymerase, based in part on genome-wide overlap of cohesin with the Negative Elongation Factor (NELF) pausing factor, and the higher levels of short promoter-proximal transcripts produced by cohesin-binding genes [12]. The PRO-seq data, which directly measures pausing, confirms these findings. The pause index is defined as the ratio of the PRO-seq signal density (normalized reads per base pair) in the 200 bp promoter region to the density in the rest of the gene body. The average pause index increases with cohesin occupancy, and the genes with the highest cohesin levels have substantially higher pausing (Figure 1F). Conversely, when active genes are divided into four groups ranging from low to high pausing (Figure S1B), the average cohesin occupancy at the promoter increases with the pause index (Figure S1C). Pausing can also be measured by the ratio of the Rpb3 ChIP signal at the promoter to the signal in the gene body [18], and this analysis also confirms that cohesin-binding genes have higher levels of pausing (Figure S1D). Although Rpb3 ChIP is not as sensitive as PRO-seq, and is not specific for transcriptionally-engaged Pol II, the concordance between the PRO-seq and Rpb3 measures of pausing agrees with the finding that most Pol II at the promoter is transcriptionally-engaged [17].

### Cohesin binds nearly all extragenic cis-regulatory modules (CRMs)

**Figure 2:**
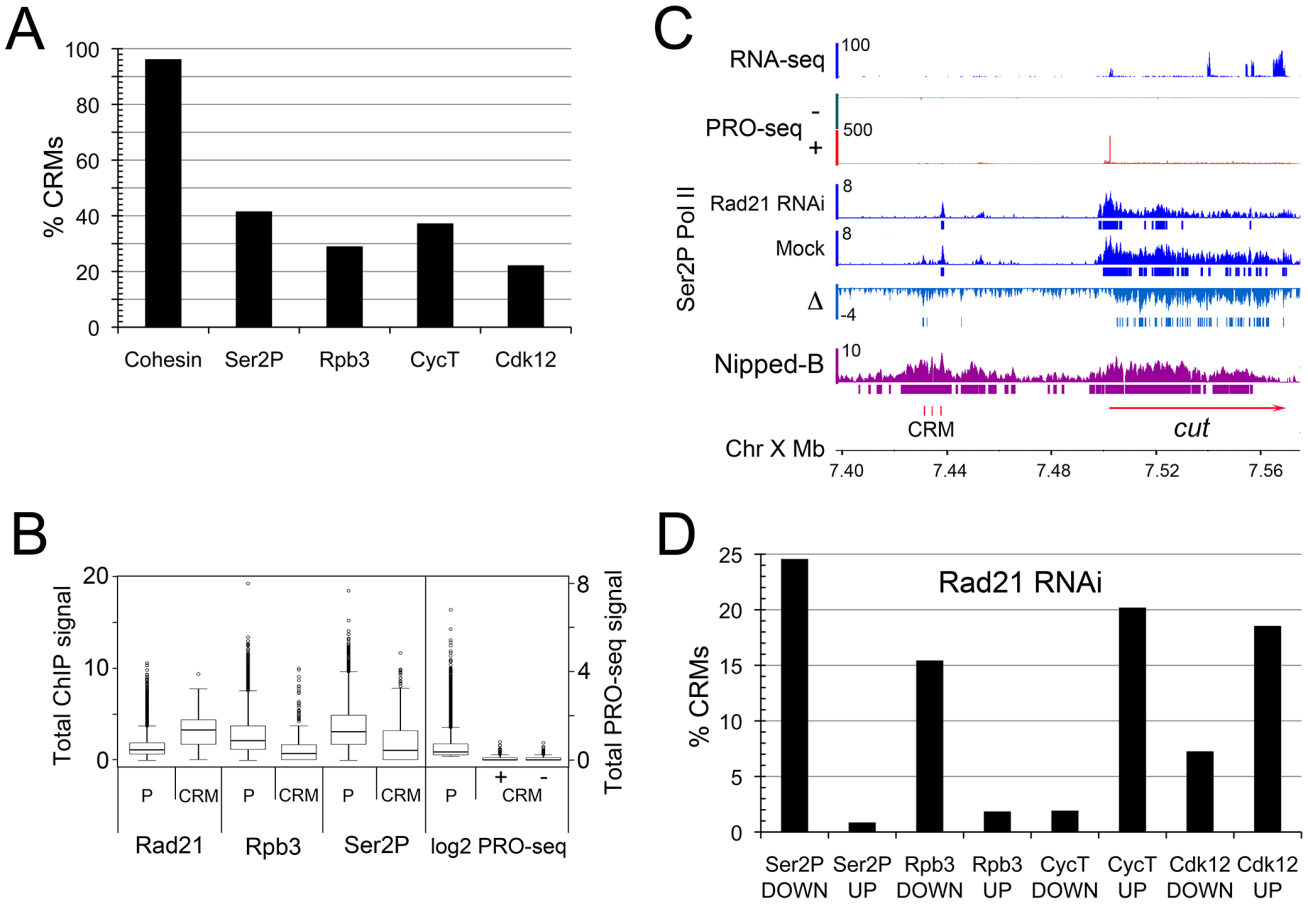
Cohesin binds nearly all predicted extragenic cis-regulatory modules (CRMs) in BG3 cells. CRMs (enhancers or other regulatory sequences) in BG3 cells were predicted from modENCODE data [22]. For purposes of determining protein occupancy and changes, all CRMs were defined as 200 bp elements centered around the DNaseI hypersensitive site (DHS). 557 putative extragenic CRMs that are a minimum of 500 bp from transcription start sites were analyzed. (A) Percent of extragenic CRMs occupied by Pol II and Pol II kinases as determined by ChIP-chip at p≤10^−3^. Cohesin binding was determined by Smc1 and Nipped-B ChIP [9]. (B) Comparison of Rad21, Rpb3, Ser2P Pol II (ChIP-chip) and PRO-seq occupancy of active promoters (P) and CRMs. The PRO-seq data is separated into + and − strands for the CRMs. (C) Example of predicted CRMs (red vertical bars) upstream of the *cut* gene. They are between the wing margin enhancer that is sensitive to Nipped-B dosage in vivo [19], [25] and other tissue-specific enhancers [24]. The PRO-seq and ChIP-chip are as described in Figure 1. RNA-seq data is from modENCODE [51]. The Ser2P Pol II Δ track is the difference in ChIP MAT score between Rad21 RNAi-treated and mock control cells. Bars below the Δ track indicate where the decrease after Rad21 depletion is ≥2 σ for ≥105 bp. (D) Percent of extragenic CRMs showing decreases (DOWN) or increases (UP) in Pol II and Pol II kinases at determined by ChIP-chip. For each protein, only those CRMs binding the protein in the control cells were used for the calculation. Decreases and increases are defined as ≥2 σ from the median genome-wide difference over a region ≥105 bp.

The Drosophila Nipped-B kollerin subunit was discovered in a genetic screen for factors that control long-range activation of the *cut* (FlyBase FBgn0004198) and *Ultrabithorax* (FlyBase FBgn0003944) genes by remote tissue-specific enhancers [19], and cohesin binds and facilitates the activity of transcriptional enhancers for pluripotency, β-globin, and T cell receptor genes in mammalian cells [6], [7], [20]. We thus examined the cohesin and Pol II occupancy of predicted extragenic cis-regulatory modules (CRMs) in BG3 cells. Active CRM/enhancer features include DNAseI hypersensitive sites (DHS), and the H3K4me1 and H3K27ac histone modifications (reviewed in [21]). The modENCODE project generated these data for BG3 cells [22], and by these criteria, there are 2,353 potential CRMs, 557 of which are not within annotated transcription units and are at least 500 bp from a transcription start site (Table S3). Forty-two of the predicted CRMs overlap 21 tissue-specific CRMs curated by the REDfly database that are functional in transgenic reporter constructs [23]. Strikingly, we find that virtually all predicted extragenic CRMs (96%) bind cohesin and Nipped-B (Figure 2A). A similar fraction (94%) of all 2,353 CRMs, which includes those located within transcribed regions, bind cohesin. Cohesin levels at the extragenic CRMs correlate positively with both the H3K27ac (r = 0.65) and H3K4me1 histone modification levels (Figure S2). Somewhat less than half of the extragenic CRMs associate with Pol II, and a similar fraction bind Pol II kinases (Figure 2A). Association of Pol II and Pol II kinases with a large fraction of these extragenic sequences supports the idea that they are functional CRMs, and the finding that virtually all bind cohesin is consistent with the idea that cohesin facilitates their function.

The average cohesin occupancy of the extragenic CRMs is higher than that for all active promoters, while the Pol II occupancy of active promoters is higher than that of the CRMs (Figure 2B). PRO-seq density indicates that much of the Pol II detected by ChIP at the CRMs is not transcriptionally engaged (Figure 2B). While the median Pol II occupancy of the predicted CRMs by ChIP is only some 3-fold lower than for promoters, the median PRO-seq density at the CRMs is indistinguishable from zero, given that less than 50% of CRMs have PRO-seq signals (Figure 2B). As seen in S2 cells [17], the mean signals at CRMs are substantially lower than those at promoters, such that the ratio of the mean PRO-seq to mean Pol II ChIP ratio is approximately 50-fold lower at CRMs than at promoters. We theorize, therefore, that most of the Pol II detected by ChIP at CRMs is promoter-bound Pol II that associates with the CRMs through DNA looping, although we cannot rule out the possibility that Pol II is directly recruited by CRM-bound proteins, but cannot initiate transcription.

Figure 2C shows clustered CRMs some 68 kb upstream of *cut*, in a region without genes, and which produces no mRNA. The surrounding region contains several enhancers that regulate the *cut* gene throughout development. The wing margin enhancer whose function is sensitive to Nipped-B dosage in vivo is 12 kb upstream of these putative CRMs, and several other tissue-specific enhancers are downstream [19], [24], [25]. The region with the CRMs contains enhancers critical for differentiation of multiple sensory cells. Gypsy transposon insulator insertions just upstream of the predicted CRMs cause primarily cut wing phenotypes, while insertions just downstream also cause head capsule defects, including deformed antenna [26].

Cohesin (Rad21) depletion substantially reduces the level of elongating Pol II on the *cut* gene as measured by Ser2P Pol II ChIP (Figure 2C), and the PRO-seq signals decrease some 40% in the gene body (Table S2). Cohesin depletion also modestly reduces the Ser2P Pol II ChIP signal in the region containing the predicted *cut* CRMs, lending support to the idea that this is a functional remote enhancer. By ChIP-chip, cohesin also influences association of Pol II with many of the other predicted extragenic CRMs around the genome. Figure 2D shows that Rpb3 and Ser2P Pol II occupancy decrease significantly on 15 to 25% of the predicted CRMs upon Rad21 depletion, consistent with the idea cohesin facilitates interactions of many CRMs with promoters.

### Cohesin and kollerin influence Pol II occupancy at many genes

Stable topological binding of cohesin to chromosomes requires loading by kollerin. Thus, depletion of cohesin and kollerin would be expected to have comparable genome-wide effects on Pol II if topologically-bound cohesin is the form that influences transcription. We compared PRO-seq measurements in mock-treated control BG3 cells to cells in which the Rad21 cohesin subunit or the Nipped-B kollerin subunit were depleted by approximately 80% using RNAi. Under these depletion conditions, there are no measurable defects in sister chromatid cohesion or chromosome segregation, and a modest decrease in the rate of cell division, which may reflect decreased expression of the Drosophila *myc* (*dm*) gene that promotes cell growth [13], [27]. These RNAi conditions reduce cohesin chromosome binding by at least 3 to 4-fold at all genes examined by ChIP, including genes that start with very high cohesin and show some of the largest changes in mRNA levels [12].

**Figure 3:**
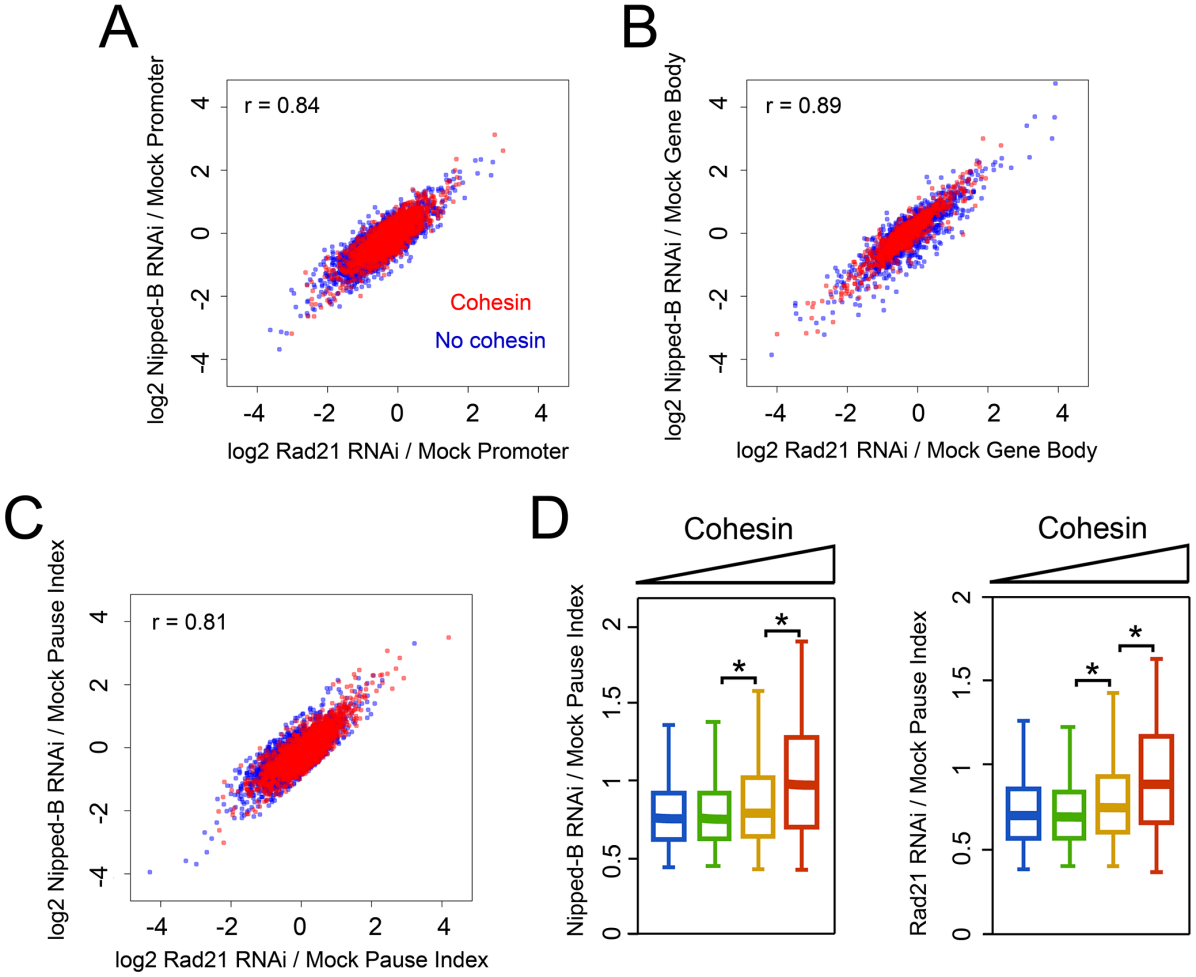
Cohesin and kollerin have very similar effects on the levels of transcriptionally engaged Pol II and promoter-proximal pausing. (A) Plot of fold-change (log2) in PRO-seq reads in the 200 bp promoter regions of PRO-seq active transcription units after Rad21 (cohesin) depletion versus Nipped-B (kollerin) depletion. The correlation between the effect of Nipped-B and Rad21 depletion is in the upper left hand corner. Genes that bind cohesin at p≤10^−3^ as determined by cohesin ChIP are in red. Genes that don't bind cohesin are in blue, many of which are hidden underneath the cohesin-binding genes. (B) Same plot for the changes in the gene bodies. (C) Same plot for changes in the pause index. (D) Fold-changes in pause index after Nipped-B (left) or Rad21 (right) depletion versus low to high (blue to red) cohesin promoter occupancy.

The effects of Rad21 and Nipped-B depletion on the PRO-seq signals in the promoter regions and gene bodies of the PRO-seq active genes are remarkably similar. The maximal changes included increases and decreases approaching 15-fold at promoters (Figure 3A), and some greater than 16-fold in gene bodies for both cohesin-binding and non-binding genes (Figure 3B). These results indicate that topologically-bound cohesin is the form that influences transcription.

### Cohesin frequently facilitates transition of paused Pol II to elongation

Cohesin and kollerin depletion also had very similar effects on the pause index, which measures the efficiency with which paused Pol II enters into elongation. Upon Rad21 or Nipped-B depletion, genes with high cohesin levels showed increased and decreased pausing at similar frequencies (Figure 3C, 3D). Thus, depletion of cohesin or the loading factor have remarkably similar effects on regulatory steps of transcription.

Overall, cohesin depletion did not substantially change the median pausing index at cohesin-binding genes, with similar numbers of genes showing increases and decreases (Figure 3D, Figure S1E). This is consistent with the prior findings that cohesin increases expression of some genes and decreases expression of others [13]. One possibility is that in addition to facilitating enhancer-promoter interactions, cohesin might also facilitate interactions of silencers that inhibit transition of Pol II to elongation. Prior studies also show that cohesin blocks transition of paused Pol II to elongation at some genes [12]. Some of these, such as *invected* and *engrailed*, are simultaneously targeted by Polycomb silencing proteins, and increase dramatically in expression upon cohesin depletion. PRO-seq confirms that such genes are among those that show the largest pausing decreases (Table S2). The presence of repressor proteins may be one factor, therefore, that determines when cohesin inhibits transition to elongation.

**Figure 4:**
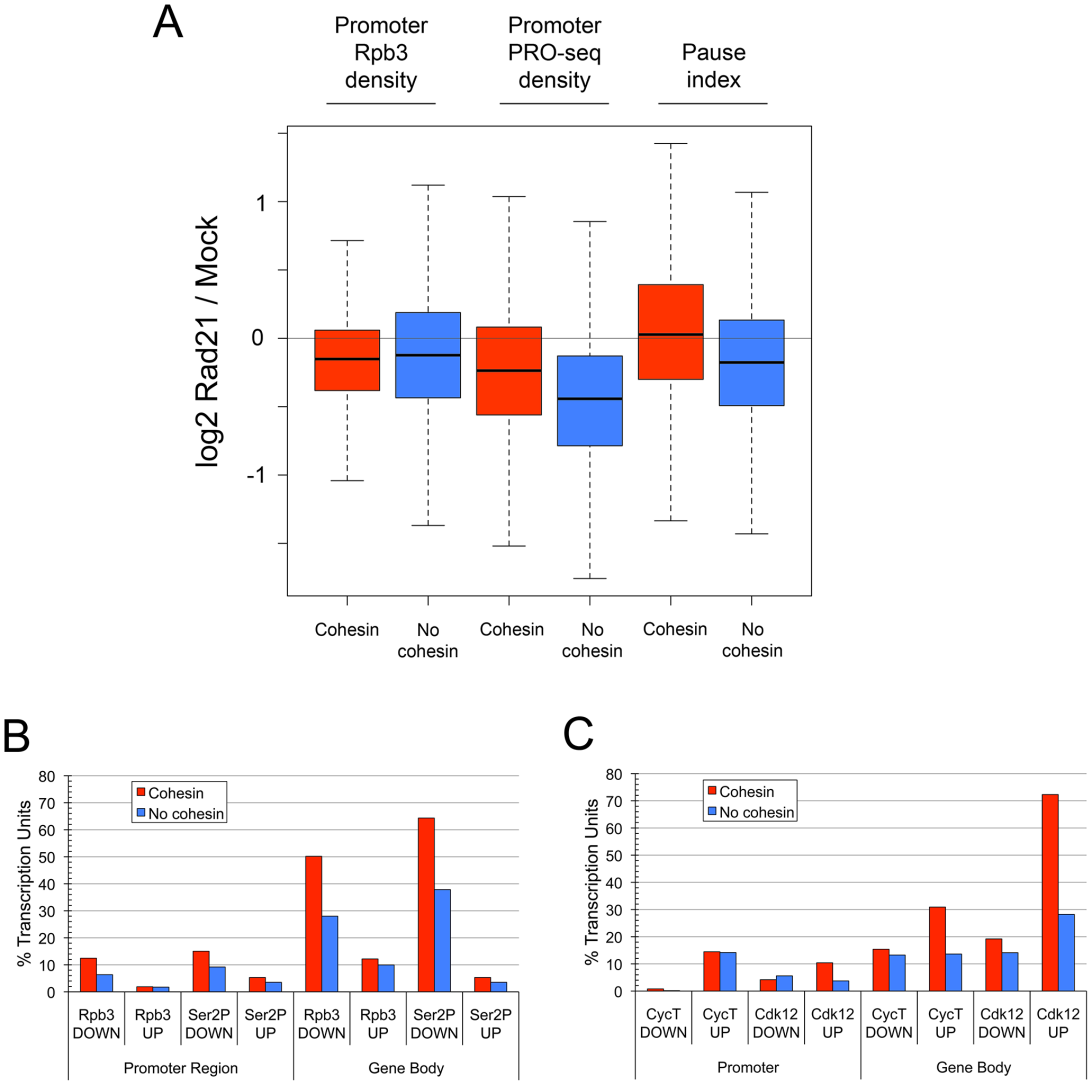
Cohesin depletion preferentially decreases transcriptional pausing and initiation at genes that lack cohesin, and increases Pol II kinase occupancy more frequently in the bodies of cohesin-binding genes. (A) The box plots show the fold changes (log2) upon Rad21 depletion for all active cohesin-binding (red) or non-binding (blue) genes in total Pol II (Rpb3 ChIP) promoter occupancy, transcriptionally-engaged Pol II (PRO-seq) at the promoter, and pausing. Cohesin-binding was called at p≤10^−3^ to distinguish cohesin-binding from non-binding genes. (B) Percent PRO-seq active cohesin-binding (red) and non-binding (blue) transcription units with absolute decreases or increases of Rpb3 (total Pol II) and Ser2P Pol II in the promoter regions and gene bodies (differences in ChIP MAT scores ≥2 σ for ≥105 bp). The higher frequencies of Rpb3 and Ser2P decreases in the bodies of cohesin binding genes compared to non-binding genes are significant (Fisher's exact test p = 1×10^−76^ and 3×10^−100^). (C) Percent PRO-seq active transcription units with decreases or increases in CycT (P-TEFb) and Cdk12 in the promoter regions and gene bodies. Only genes binding CycT or Cdk12 at p≤10^−3^ in control cells were used for this analysis. The differences in CycT and Cdk12 increases in the bodies of cohesin-binding versus non-binding genes are significant (p = 2.6×10^−18^ and 9.6×10^−68^).

Unexpectedly, cohesin depletion indirectly reduced pausing at most genes that lack cohesin, with a median decrease of 25% (genes in lowest cohesin group in Figure 3D). In control cells, the median pause index at the genes with the highest cohesin levels is 3.7-fold higher than at the genes without cohesin (Figure 1F). However, cohesin depletion increases this ratio to 8.7, primarily because of the broad decrease in pausing at genes that lack cohesin. The overall reduction in pausing might suggest that pausing factors are diminished, but the mRNA levels for all NELF and DSIF subunits are virtually unaffected by cohesin or Nipped-B depletion [13]. Both cohesin-binding and non-binding genes show frequent decreases in promoter PRO-seq density, but these decreases are substantially more frequent at genes that lack cohesin, which likely explains why they also show more frequent decreases in the pause index (Figure 4A). If this indirect general pausing decrease caused by cohesin depletion also occurs at cohesin-binding genes, then it will counteract and obscure many direct increases in pausing caused by cohesin depletion. If so, it can be inferred that cohesin directly facilitates transition to elongation even more frequently than the raw data indicates.

By facilitating enhancer-promoter contact, cohesin could increase the rates of distinct steps of transcription: Pol II recruitment, transcription initiation, or the transition of paused Pol II to elongation. In addition, cohesin bound at the promoters of cohesin-binding genes could directly influence all three steps. The finding that cohesin depletion reduces promoter PRO-seq density less frequently at cohesin-binding genes than at genes that lack cohesin (Figure 4A) argues that recruitment or initiation are less often directly influenced by cohesin. Strikingly, although PRO-seq density is more frequently decreased at the promoters of genes that lack cohesin, there is little difference in the overall effect of cohesin depletion on total Pol II occupancy at cohesin-binding and non-binding promoters as measured by Rpb3 ChIP, further supporting the idea that Pol II recruitment is not usually directly affected by cohesin (Figure 4A). This predicts that genes that lack cohesin would not show as dramatic pausing decrease upon cohesin depletion if pausing was calculated using Rpb3 ChIP instead of PRO-seq data, which was confirmed (Figure S1E). Because the average decrease in PRO-seq at the promoters that lack cohesin is greater than that at cohesin-binding promoters, but the average change in total Pol II occupancy is similar, we deduce that transcription initiation is frequently reduced at genes that lack cohesin.

The more frequent increase in transcriptional pausing at cohesin-binding genes relative to genes that lack cohesin in response to cohesin depletion predicts that cohesin more often directly facilitates transition of paused polymerase to elongation at many genes. To confirm this idea, we compared the frequency of absolute changes in Pol II occupancy of promoters and gene bodies caused by cohesin depletion using the genomic ChIP data for Rpb3 and Ser2P Pol II. We set a statistical threshold for increases or decreases (see Materials and Methods) to determine how many promoters and gene bodies show significant changes upon cohesin depletion. This revealed that total or Ser2P Pol II occupancy rarely increased at the promoters or in the bodies of either cohesin-binding or non-binding genes upon cohesin depletion (Figure 4B). Decreases in Pol II at the promoters were also rare, but more frequent than increases. Cohesin depletion caused significant absolute decreases in Rpb3 and Ser2P Pol II in the bodies of more than half of the cohesin-binding genes, almost twice as often as in genes that lack cohesin (Figure 4B). We conclude, therefore, that cohesin often directly increases transition of paused Pol II to elongation, and less frequently directly influences Pol II recruitment or transcriptional initiation.

Although infrequent, absolute reductions in total Pol II promoter occupancy after cohesin depletion that met the statistical threshold were detected twice as often at cohesin-binding genes than at genes that lack cohesin (Figure 4B). This is still consistent with the finding that the average fold-changes in total Pol II promoter occupancy at cohesin-binding and non-binding genes are similar (Figure 4A), because cohesin-binding genes have higher levels of Pol II at the promoter (Figure 1B). The same absolute change in Pol II occupancy would therefore be a smaller fold-change at most cohesin-binding genes than at most genes that lack cohesin.

We suspect that the reduced pausing that reflects reduced transcription initiation at most genes that lack cohesin is caused by altered expression of factors that act broadly at many or all genes, such as basal transcription factors. Cohesin depletion, however, does not significantly reduce expression of known basal factors such as TFIIB [13]. Prior work has shown that cohesin directly promotes *dm/myc* expression, and the global pattern of decreases in mRNA upon depletion of cohesin in BG3 cells strongly overlaps those seen in *dm/myc* mutants [13], [27], [28]. Thus another possibility, consistent with the recent reports that Myc directly amplifies transcription of most if not all active genes in a variety of mammalian cell types [29], [30], is that reduced *dm/myc* expression could contribute to the broad indirect effect of cohesin depletion on most genes that lack cohesin.

### Cohesin influences the distribution of the P-TEFb and Cdk12 Pol II kinases

The P-TEFb Pol II kinase, which can be recruited by transcriptional activator proteins bound to enhancers or promoters, stimulates transition of paused Pol II to elongation by phosphorylating NELF, DSIF, and the C-terminal domain of the large subunit of Pol II (reviewed in [31]). Cdk12 is also responsible for a large fraction of Ser2P Pol II phosphorylation [32]. We tested the idea that cohesin promotes transition of paused Pol II to elongation by facilitating recruitment of P-TEFb or Cdk12 by comparing the CycT and Cdk12 ChIP signals in control cells and cells in which cohesin was depleted. We restricted the analysis to those genes in which CycT or Cdk12 was detected in the control cells, to make it possible to detect both decreases and increases. Surprisingly, after cohesin depletion, decreases in CycT or Cdk12 in any transcription units are very rare (Figure 4C). Indeed, CycT and Cdk12 both increase more frequently at promoters and gene bodies than they decrease upon cohesin depletion, and more than twice as often in the bodies of cohesin-binding than in non-binding genes (Figure 4C). Similar frequencies of CycT and Cdk12 increases are seen when all active genes are scored, indicating that increases also occur when the kinases are not detected prior to cohesin depletion. These increases are generally modest, but usually occur in genes with Ser2P Pol II decreases, and are strong enough to give up to a 1.5-fold increase in ratios of the kinases to total Pol II in the bodies of cohesin-binding genes (Figure S3). Because there are several heptapeptide repeats in Pol II, a decrease in the fraction of heptapeptide repeats that are phosphorylated within each Rpb1 molecule could increase the net number of unmodified sites available for kinase binding, even with a decrease in the level of Pol II in the gene body. Based on these findings we conclude that the frequent reduction in phosphorylated Pol II in gene bodies upon cohesin depletion is not caused by reduced presence of the Pol II kinases, and theorize instead that cohesin may facilitate efficient modification of Pol II.

### Cohesin-binding genes produce more steady state mRNA per transcribing Pol II complex

**Figure 5:**
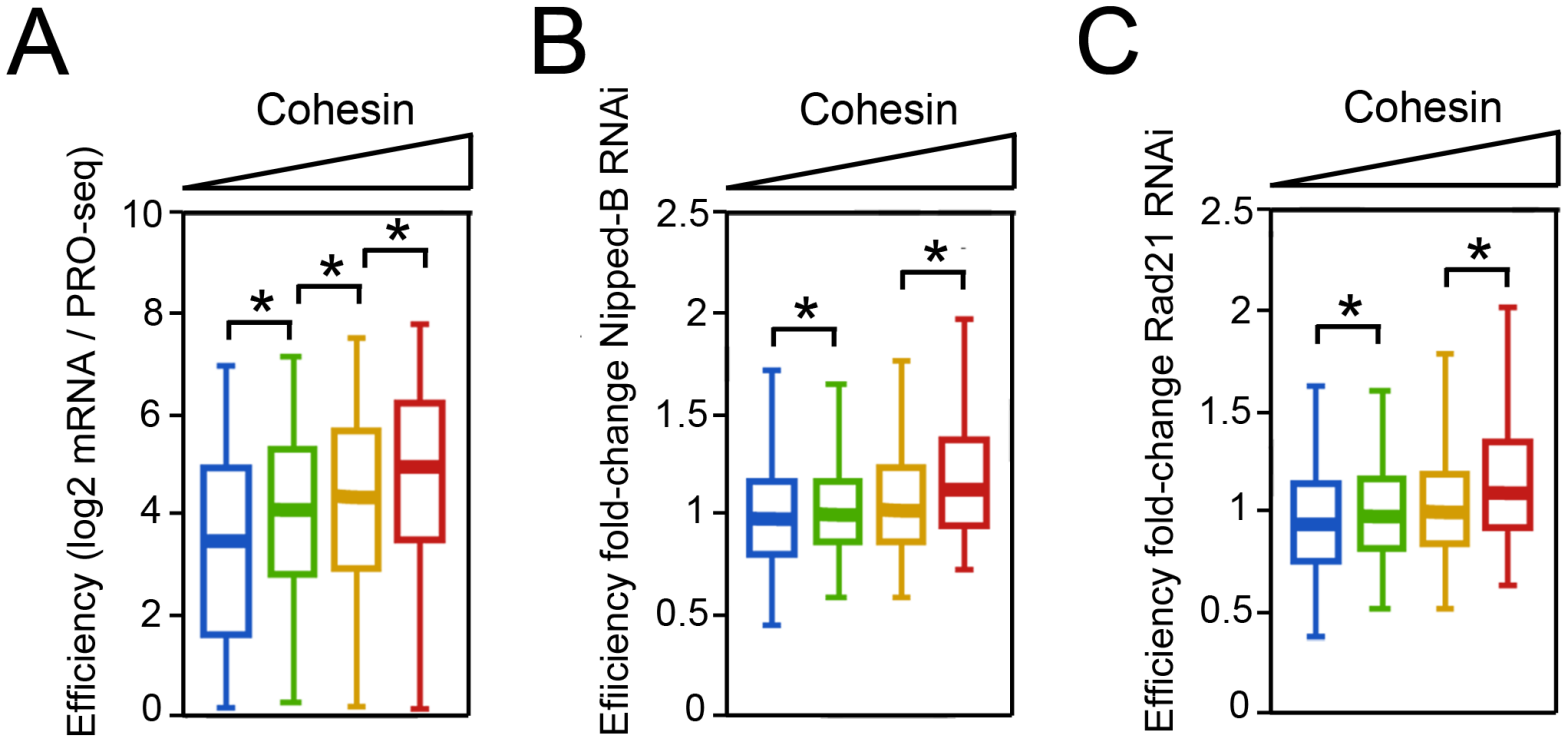
Cohesin-binding genes produce more steady state mRNA per elongating Pol II complex in BG3 cells. (A) The efficiency (log2 mRNA/Gene Body PRO-seq) of genes with different levels of cohesin at the promoter. (B) The fold-change in efficiency upon kollerin (Nipped-B). (C) The fold change in efficiency upon cohesin (Rad21) depletion.

The higher Pol II occupancy of cohesin-binding genes predicts that they should produce more mRNA on average, assuming that RNA processing, transport and stability do not differ substantially between cohesin-binding and non-binding genes. To test this idea, we used existing mRNA measurements [13] to calculate the ratio of steady-state mRNA to PRO-seq density in the gene body, which we define as “efficiency”. This surprisingly revealed that the average efficiency increases significantly with the cohesin level, and that the genes with the highest cohesin levels produce some 2-fold more steady-state mRNA per transcribing Pol II complex than genes that lack cohesin (Figure 5A).

Cohesin is not responsible for the higher efficiency. Upon Nipped-B or Rad21 depletion, the average efficiency of the genes with the highest cohesin levels actually increases modestly (Figure 5B, 5C). We currently do not know why cohesin-binding genes are more efficient, but note that they are highly transcribed, lack histone H3 lysine 36 trimethylation (H3K36me3), and are highly enriched for UG repeats in the nascent transcripts [12]. H3K36me3 and UG repeats regulate RNA processing, and binding of the TDP-43 protein to UG repeats stabilizes long nascent transcripts and reduces missplicing in mammalian neural tissue [33]–[36].

## Discussion

### Cohesin directly influences transcription and transition of paused Pol II to elongation

**Figure 6:**
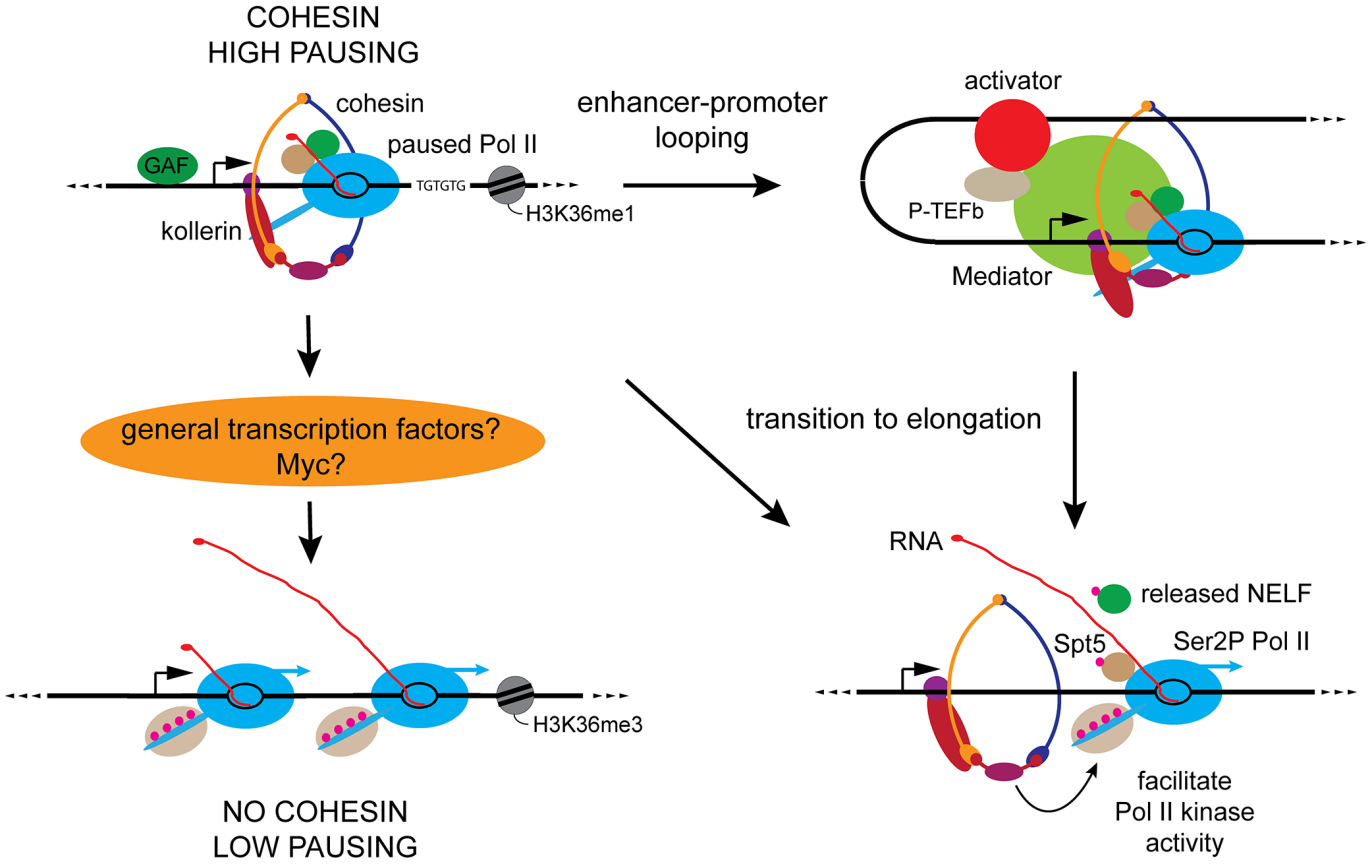
Key features of cohesin-binding genes and proposed roles for cohesin in genome-wide control of Pol II activity. Cohesin-binding genes (upper left) have higher levels of Pol II and promoter-proximal Pol II pausing than other active genes (lower left). Cohesin-binding genes are enriched for GAGA factor (GAF) binding upstream of the promoter and TG repeats in the transcribed region, and unlike other active genes, lack the H3K36me3 histone modification [12]. Current findings indicate that cohesin facilitates looping and contact between enhancers and promoters (upper right), which primarily facilitates transition of paused Pol II to elongation. Cohesin at the promoter may also directly facilitate transition to elongation by increasing the efficiency of P-TEFb or Cdk12 phosphorylation of Pol II and the Spt5 and NELF pausing factors (lower right). To explain the broad effect on Pol II activity at genes that don't bind cohesin, we hypothesize that cohesin promotes expression of broadly acting transcription factors such as Myc that regulate many or most genes (lower left).

These studies provide compelling evidence that cohesin directly influences transcription. Comparing the effects of cohesin depletion on Pol II occupancy and activity shows that on average, cohesin-binding genes respond differently to cohesin depletion than non-binding genes, allowing us to infer that cohesin directly influences Pol II occupancy and activity at genes that it binds. This direct influence is likely mediated by facilitating looping interactions with enhancers, and also direct effects on the transition of paused Pol II to elongation at the promoter (Figure 6).

Beyond the generally higher levels of Pol II, the most remarkable differences between cohesin-binding and non-binding genes are in promoter-proximal transcriptional pausing. Cohesin-binding genes have a substantially higher average pausing index, and are much more likely than non-binding genes to show increased pausing upon cohesin depletion. Coupled with the decreases in Ser2P Pol II in the bodies of most cohesin-binding genes, the increased pausing upon cohesin depletion argues that cohesin facilitates the transition of paused polymerase to elongation at many genes that it binds.

Cohesin can increase the rate of Pol II transition to elongation by facilitating enhancer-promoter looping, which would bring transcriptional activators and the P-TEFb they recruit into contact with the paused Pol II to stimulate transition to elongation (Figure 6). Indeed, genetic evidence from Drosophila and chromosome conformation capture (3C) data from mammalian cells supports the idea that cohesin facilitates communication and looping between enhancers and promoters [6], [7], [19], [20]. In mammals, cohesin is present at the extragenic enhancers for several mammalian pluripotency genes, the *β-globin* gene and the T cell receptor locus, and at many CRMs defined by the binding of multiple tissue-specific transcription factors [6], [7], [20], [37]. In Drosophila BG3 cells, cohesin occupies essentially all CRMs, and the reduced Pol II occupancy at many upon cohesin depletion further expands the idea that enhancer-promoter communication is one of cohesin's key roles at several genes. Several studies indicate that cohesin also facilitates looping between sites binding the CTCF protein in mammalian cells to regulate gene expression, but this function is not conserved in Drosophila (reviewed in [1], [2]).

Many studies support a role for enhancers in the assembly of pre-initiation complexes at promoters, but also indicate that they can control other steps, including the transition of Pol II at the promoter to elongation [21]. The steps in activation controlled by a particular enhancer likely depend on the constellation of enhancer-bound transcription factors. If an enhancer's main function is pre-initiation complex formation, then we would expect to see frequent Pol II decreases at promoters upon cohesin depletion. Such decreases, however, are actually infrequent compared to gene body decreases in our experiments. Our data suggest, therefore, that once a gene is active, the primary function of most enhancers is to stimulate paused Pol II to enter elongation.

The analysis presented here cannot definitively address to what extent reduced enhancer-promoter communication explains Pol II decreases in the bodies of cohesin-binding genes caused by cohesin depletion. A critical limitation is that we do not yet know all the contacts between enhancers and promoters, and whether such contacts are cohesin-dependent. We note, however, that the high levels of cohesin at promoters, including at many genes that likely lack enhancers, raises the possibility that cohesin directly interacts with the paused Pol II complex and influences the transition to elongation. These interactions may involve increasing the efficiency with which P-TEFb and Cdk12 modify Pol II or the NELF and DSIF pausing complexes (Figure 6). We suggest that cohesin is more critical for kinase efficiency than for kinase recruitment because at most genes where cohesin depletion reduces Pol II phosphorylation, the kinase level in the gene body actually increases. Also consistent with the idea that promoter-bound cohesin directly influences transition to elongation is the finding that cohesin interacts with the Mediator complex [7]. In addition to facilitating assembly of the pre-initiation complex, Mediator is implicated in recruitment of elongation factors and efficient transcriptional elongation post-initiation [38]–[40].

The idea that promoter-bound cohesin directly influences transition of Pol II to elongation is also supported by prior work showing that cohesin inhibits transition to elongation at several cohesin-repressed genes [12]. In those studies, cohesin and pausing factor depletion experiments revealed that cohesin inhibits transition of Pol II to elongation at a step distinct and likely downstream from those controlled by the NELF and DSIF pausing factors. This inhibition is unlikely to be physical obstruction of Pol II movement because cohesin depletion did not increase the rate of elongation along the induced *EcR* gene. Moreover, many of the cohesin-repressed genes are among the rare cohesin-binding genes targeted by the PRC2 Polycomb group silencing complex. Thus the presence of repressor proteins may be one factor that determines whether promoter-bound cohesin facilitates or inhibits transition to elongation. Many cohesin-repressed genes are those that show the largest increases in mRNA upon cohesin depletion [13], and more Pol II in the gene bodies in this study. In general, these cohesin-repressed genes show little or no change in Pol II occupancy at the promoter upon cohesin depletion, further supporting the idea that repression largely reflects inhibition of entry into elongation and not Pol II recruitment [12, this study].

### How does cohesin depletion alter Pol II activity at most genes that don't bind cohesin?

We unexpectedly observed that cohesin depletion reduces promoter-proximal Pol II pausing at most genes that don't bind cohesin. Cohesin depletion does not alter expression of genes encoding subunits of the NELF and DSIF pausing factors or the Pol II kinases, and very modestly increases expression of some Mediator subunit genes [13]. The reduction in transcriptionally-engaged Pol II at the promoter measured by PRO-seq is also more significant than the effect on total Pol II occupancy at genes that lack cohesin. We theorize, therefore, that cohesin controls expression of factors that operate broadly to facilitate transcription initiation.

The key suspects for general factors controlled by cohesin are general basal transcription factors, or possibly Diminutive (Dm), the Drosophila Myc protein (Figure 6). Cohesin depletion does not significantly decrease the mRNAs that encode the known basal transcription factors, but does substantially reduce *dm/myc* transcription. Recent studies in mammalian cells show that Myc directly amplifies transcription of most active genes [29], [30] and therefore reduction of *dm/myc* expression upon cohesin depletion is expected to alter transcription of many genes, including those that do not bind cohesin. The mammalian studies also indicate, however, that chemical ablation of Myc function increases pausing at Myc target genes [29], [30], [41], while our PRO-seq measurements argue that pausing generally decreases upon cohesin depletion. The mammalian experiments measured pausing by Pol II ChIP, which does not distinguish between promoter-bound Pol II that is transcriptionally-engaged from Pol II that has not initiated transcription, or is somehow otherwise blocked from elongation. In our experiments, Pol II ChIP did not show the same pausing decrease as PRO-seq upon cohesin depletion. Thus, although Myc appears to function as an anti-pausing factor, we cannot rule out the possibility that reduced *dm/myc* expression is responsible for many of the indirect effects of cohesin depletion on transcription initiation. Direct positive regulation of *myc* by cohesin occurs in Drosophila, zebrafish, mice and humans [8], [13], [27], [42]. As a key regulator of growth and protein synthesis, it is likely that reduced *myc* expression contributes to the poor growth of individuals with Cornelia de Lange syndrome and *Nipbl*(+/−) mice [42], [43].

### Why do cohesin-binding genes more efficiently produce mRNA?

Based on their higher Pol II occupancy, we expected that cohesin-binding genes would produce more mRNA on average, in proportion to the Pol Il levels. We observed, however, that they produced disproportionately more steady-state mRNA per transcriptionally-engaged Pol II complex, with the genes that have high cohesin levels being twice as efficient as the genes that lack cohesin. Cohesin depletion did not reduce the efficiency, indicating that these genes have other features that make them more efficient. Prior studies show that cohesin-binding genes lack the H3K36me3 histone modification, which is found on other active genes, and is mediated by the Set2 protein that travels with the phosphorylated C terminal domain of the Rpb1 Pol II subunit [44]. H3K36me3 influences RNA processing and vice versa [34], [35]. We currently favor the idea, therefore, that co-transcriptional RNA processing, which also affects RNA transport and stability, is more efficient at cohesin-binding genes. Alternatively, elongation rates, which can be influenced by the higher Pol II density at these genes, may be higher. Cohesin-binding genes are also highly enriched for TG repeats in transcribed plus-strand non-coding sequences 50 to 800 bp downstream of the promoter, and thus the nascent RNAs contain UG repeats [12]. One factor that binds UG repeats is TDP-43 (TBPH in Drosophila), which influences RNA processing, and increases the stability of many long nascent RNAs and splicing fidelity in mouse brain [33], [36]. It is possible that these repeats also participate in cohesin recruitment, which could explain the correlation between cohesin-binding and high efficiency.

## Materials and Methods

### Cell culture and RNAi depletion of Rad21 and Nipped-B

Culture of ML-DmBG3-c2 (BG3) cells and RNAi depletion of Nipped-B and Rad21 were conducted as previously described [13].

### ChIP–chip

Genomic chromatin immunoprecipitation of RNAi-treated and mock-treated BG3 cells was performed using Affymetrix Drosophila 2.0R genome tiling arrays as previously described [9] except chromatin sonication was performed under standardized conditions with a Diagenode Bioruptor, and precipitated DNA was amplified using commercial Whole Genome Amplification reagents (Sigma-Aldrich). Reverse-crosslinked chromatin was used to prepare probes for input control arrays. All ChIP-chip data generated for this study is the average of two independent biological replicates. Karen Adelman (NIEHS) provided Rpb3 antibodies, Akira Nakamura (Riken, Japan) provided CycT antibodies, and Bart Bartkowiak and Arno Greenleaf (Duke) provided Cdk12 antibodies. Ser2P Pol II antibodies were purchased from Abcam (ab5095). The Drosophila Rpb3 antibody has been previously been validated for ChIP-chip [18]. The Ser2P Pol II antibody was previously validated for specificity in Drosophila by in vivo inactivation of P-TEFb by the Pgc protein followed by immunostaining and western blots [45]. We retested the Ser2P Pol II antibody by treating BG3 cells with flavopiridol to inhibit P-TEFb followed by western blotting and observed that the major band decreases in intensity over time, although there is an unaffected minor band that co-migrates with the unmodified Rpb1 detected by the 8WG16 antibody (Figure S4). The Cdk12 antibody has previously been validated for ChIP [32]. The Drosophila CycT antibody was previously validated [45] and in our tests, it recognizes a single major protein of the expected size in western blots of whole cell extracts that is reduced by CycT RNAi treatment (Figure S5).

MAT software [46] was used to calculate ChIP enrichment across the Drosophila genome. MAT performs within-array normalization using individual probe DNA sequences, and MAT scores measure enrichment relative to an input control. MAT scores scale linearly with log2 IP/control enrichment values as determined by processing the same data with TiMAT (http://bdtnp.lbl.gov/TiMAT/). MAT is the optimal algorithm for analysis of Affymetrix array ChIP-chip, and provides peak detection sensitivity equivalent to ChIP-seq performed at a density of one read per genome base pair [47], [48]. ChIP-chip data has been deposited in the GEO database (accession no. GSE42399).

### PRO-seq

Precision global run-on sequencing for control cells, and cells depleted for Rad21 and Nipped-B, was conducted as described elsewhere [15], except that a simplified cell permeabilization nuclear isolation protocol was used. All steps were conducted at 4° unless indicated otherwise. 2.5 to 7.5×10^8^ control or RNAi-treated BG3 cells were collected by centrifugation (1000 g for 5 min), suspended in 5 to 10 mL Phosphate Buffered Saline (PBS) pH 7.0, collected by centrifugation, suspended in 5 mL Buffer W [10 mM Tris-HCl pH 7.5,10 mM KCl, 150 mM sucrose 5 mM MgCl_2_, 0.5 mM CaCl_2_, 0.5 mM dithiothreitol (DTT)], and collected by centrifugation. The cells were suspended in 5 mL Buffer P (10 mM Tris-HCl, pH 7.5, 10 mM KCl, 250 mM sucrose, 5 mM MgCl_2_, 1 mM EGTA, 0.05% Tween-20, 0.5 mM DTT), the suspension was adjusted to 0.14% NP-40, and then incubated on ice for 3 min. The nuclei were washed twice in 5 mL Buffer W, suspended in 1 mL Buffer W, and transferred to a siliconized 1.5 mL microcentrifuge tube. The nuclei were collected by centrifugation at 1000 g for 5 min, suspended in 0.5 mL Buffer F (50 mM Tris-HCL pH 8.3, 40% glycerol, 5 mM MgCl_2_, 0.1 mM EDTA, 0.5 mM DTT), and counted using a hemacytometer. The nuclei were then suspended in Buffer F to concentration of 40 to 50×10^5^ per microliter, distributed into 100 microliter aliquots in siliconized 1.5 mL tubes, snap frozen in liquid nitrogen, and stored at −80°. The PRO-seq data has been deposited in the GEO database (accession no. GSE42399).

### Data analysis

PRO-seq reads for each duplicate sample were summed over the promoter regions and gene bodies of nearly 17,000 annotated transcription units and normalized to the total reads for each sample. Mathematical and statistical analysis of the samples was conducted using Microsoft Excel, R ([49], http://www.R-project.org), and custom programs. After confirming high correlations between the duplicate samples (Table S1), the values for the two duplicates for each condition (Mock, Rad21 depleted, Nipped-B depleted) were averaged (Table S2). PRO-seq active genes were defined as those in which there were an average of at least 1 read per million in both the 200 bp promoter region and the gene body in control samples. PRO-seq changes in the promoter regions, gene bodies, and pausing index upon cohesin depletion were calculated and plotted using R. To rank genes according to cohesin-binding levels, the Rad21 ChIP-chip MAT scores over the promoter regions of PRO-seq active genes were integrated, and the genes broken into four categories ranging from low to high mean cohesin levels, using a geometric distribution (Figure 1A, lower right panel). The lowest group had mean ChIP MAT scores between 0 to 1 in the 400 bp region surrounding the transcription start site, the next highest group had mean scores between 1 to 2, then 2 to 4 and the highest group was greater than 4. This method allowed finer distinction between cohesin-binding levels than quartiles.

To measure the fraction of PRO-seq active genes or putative CRMs that bind or do not bind cohesin (Rad21, Smc1, Nipped-B), bed files showing binding of Rpb3, Ser2P Pol II and CycT at p≤10^−3^ were generated using MAT software. Binding of Rpb3, Ser2P Pol II and CycT to PRO-seq active genes was determined using BEDTools software [50] to detect overlaps of the bed files with 200 bp promoter regions and gene bodies of PRO-seq active transcription units, and putative active enhancers, with a 1 bp minimum overlap. Existing Smc1 and Nipped-B ChIP-chip data for BG3 cells ([9], GEO accession no. GSE9248) was used to determine which genes and putative enhancers bind cohesin.

For some analyses, the differences in the ChIP enrichment (MAT scores) for Pol II or Pol II kinases were calculated at each of the nearly 2.8 million points measured across the genome. The distributions of the differences, means, medians and standard deviations of the differences were determined using R. In all cases, there was minimal skew in the distribution of differences, and both the mean and median differences were nearly identical and close to zero. The thresholding tool of the Affymetrix Integrated Genome Browser (IGB; http://www.affymetrix.com/partners_programs/programs/developer/tools/download_igb.affx) was used to generate bed files indicating where the Rad21 RNAi sample enrichment differs from the enrichment in control cells by at least two standard deviations from the median genome-wide difference over at least 105 bp (three microarray features, example in Figure 2). BEDTools was used to detect overlaps between these intervals and the 200 bp promoter regions or gene bodies of the PRO-seq active genes, or predicted extragenic CRMs to identify those with significant changes. The rare instances in which a feature scored positive for both a decrease and an increase in ChIP signal were resolved by visual inspection. In most cases these reflect both a small increase and a small decrease, and the genes were rescored as having no significant change. This method agrees with changes in Pol II occupancy after Rad21 depletion previously measured at multiple genes by quantitative real-time PCR ChIP in independent experiments [12].

To measure mRNA production efficiency we used expression data for 13,132 genes in BG3 cells previously measured by Affymetrix Drosophila GeneChip 2.0 for mRNA levels in control and Rad21 depleted BG3 cells ([13], GEO accession no. GSE16152). For those genes represented by multiple probes, we summed the total signals for all probes, and used the total to compare to the gene body PRO-seq signals.

## Supporting Information

Figure S1 Cohesin binding genes have higher promoter-proximal pausing and Pol II occupancy. (A) Cohesin-binding groups of active genes used in Figure 1 based on levels of cohesin at the promoter. The numbers above or below each box plot indicate the number of genes in each group. (B) PRO-seq active genes distributed into groups based on pause index. (C) Cohesin (Rad21) occupancy of genes with increasing pausing. (D) Pause index of cohesin-binding groups calculated using ChIP-chip data [mean Rpb3 ChIP signal at promoter (P) divided by mean ChIP signal in gene body (GB)]. (E) Fold-change in pause index of cohesin-binding groups measured by Rpb3 ChIP upon Rad21 deletion. (F) Percent of transcription units with Rpb3, Ser2P Pol II, CycT (P-TEFb), and Cdk12 ChIP signal in the 200 bp promoter region surrounding the annotated transcription start site and the gene body at p≤10^−3^. The genes were divided in cohesin-binding and non-binding by Smc1 and Nipped-B occupancy [9] of the promoter region at p≤10^−3^.
(TIF)

Figure S2 Cohesin binding correlates with the H3K27ac and H3K4me1 histone modifications at extragenic cis regulatory modules (CRMs). The box plots in the upper left panel show the distributions of the cohesin, H3K27ac and H3K4me1 ChIP signals at 557 extragenic CRMs (Table S3). The remaining panels plot the indicated ChIP signals against each other at each extragenic CRM and give the corresponding correlation coefficients.
(TIF)

Figure S3 Cohesin depletion increases Cdk12 occupancy in gene bodies that have decreased phosphorylated Pol II. (A) Overlap of cohesin-Cdk12 binding genes with decreases in Ser2P Pol II in the gene body and increases in Cdk12. “Other” indicates no significant changes, an increase in Ser2P Pol II, or a decrease in Cdk12. (B) Examples of genes with Ser2P Pol II decreases in the gene body and an increase in Cdk12 and/or CycT. The top tracks show the PRO-seq reads in the control cells. The PRO-seq scale is 1000 for *arm*, *bab2* and *pnt*, and 4000 for *aop*. The Δ tracks below the ChIP tracks show the difference in MAT score between the Rad21 depleted and control cells. The bars above and below the Δ tracks show where increases and decreases are ≥2 σ for regions ≥105 bp. (C) The left panel shows the Rad21 (cohesin) levels for the genes used in this analysis. They are three of the four groups shown in Figure S1A. The kinase ChIP signals in the group with the lowest cohesin binding were often too low to generate reliable fold-change ratios and kinase to Pol II ratios. The middle and right panels show the fold-change in the ratio of Cdk12 and CycT to Rpb3 in the gene body for each of the three cohesin-binding groups.
(TIF)

Figure S4 Validation of Ser2P Pol II antibody. The panels show a western blot of whole cell extracts of BG3 cells mock treated or treated with 1 µM flavopiridol, a P-TEFb inhibitor. The top panel shows the signal obtained with Ser2P Pol II antibody (Abcam ab5095) diluted 1∶1000. The middle panel is the same western reprobed with the 8WG16 antibody that recognizes primarily non-phosphorylated Rpb1, and the bottom panel shows the same blot probed with anti-actin as an internal standard. The asterisk (*) indicates a band that co-migrates with non-phosphorylated Rpb1, indicating that ab5095 antibody may slightly cross-react with non-phosphorylated Rpb1.
(TIF)

Figure S5 Validation of CycT antibody. BG3 cells were mock treated (M) or treated with one of two different dsRNAs (1 and 2) against with CycT for 3 days. Templates for synthesis of dsRNA were made by PCR from genomic DNA and dsRNA was prepared as previously described [13]. The PCR primers used to make template for dsRNA 1 were 5′-TAATACGACTCACTATAGGGAGACTCTTCCCAATGAGCCTCTG-3′ and 5′-TAATACGACTCACTATAGGGAGACATGGATGGTGGTACAGCAG-3′, and for dsRNA 2 5′-TAATACGACTCACTATAGGGAGACAAGCTAAATAGCCATCCGC-3′ and 5′-TAATACGACTCACTATAGGGAGAGGCGTGTGTTTCTCCTCAT-3′. Proteins were extracted from cells with buffer (10 microliters per ∼5×10^5^ cells) containing 40 mM Tris-HCl pH 7.4, 8 M urea, and 1% NP-40. After SDS-PAGE (∼2.5×10^5^ cells per lane) on a 4–20% gradient gel (Biorad TGX), proteins were electrotransferred to Immobilon-P membrane in buffer contain 100 mM CAPS pH 10.8 and 10% methanol. The western blot was probed with a 1∶1000 dilution of the CycT rabbit antiserum [45].
(TIF)

Table S1 PRO-seq sequencing statistics.
(DOCX)

Table S2 Average PRO-seq promoter (P) and gene body read densities in annotated transcription units.
(XLSX)

Table S3 Predicted extragenic cis-regulatory modules (CRMs).
(XLSX)
