# Supplementary figures and images for: Genome-Wide Control of RNA Polymerase II Activity by Cohesin
Source: PLoS Genet. 2013 Mar 21;9(3):e1003382. doi: 10.1371/journal.pgen.1003382 (PMC3605059; doi:10.1371/journal.pgen.1003382)

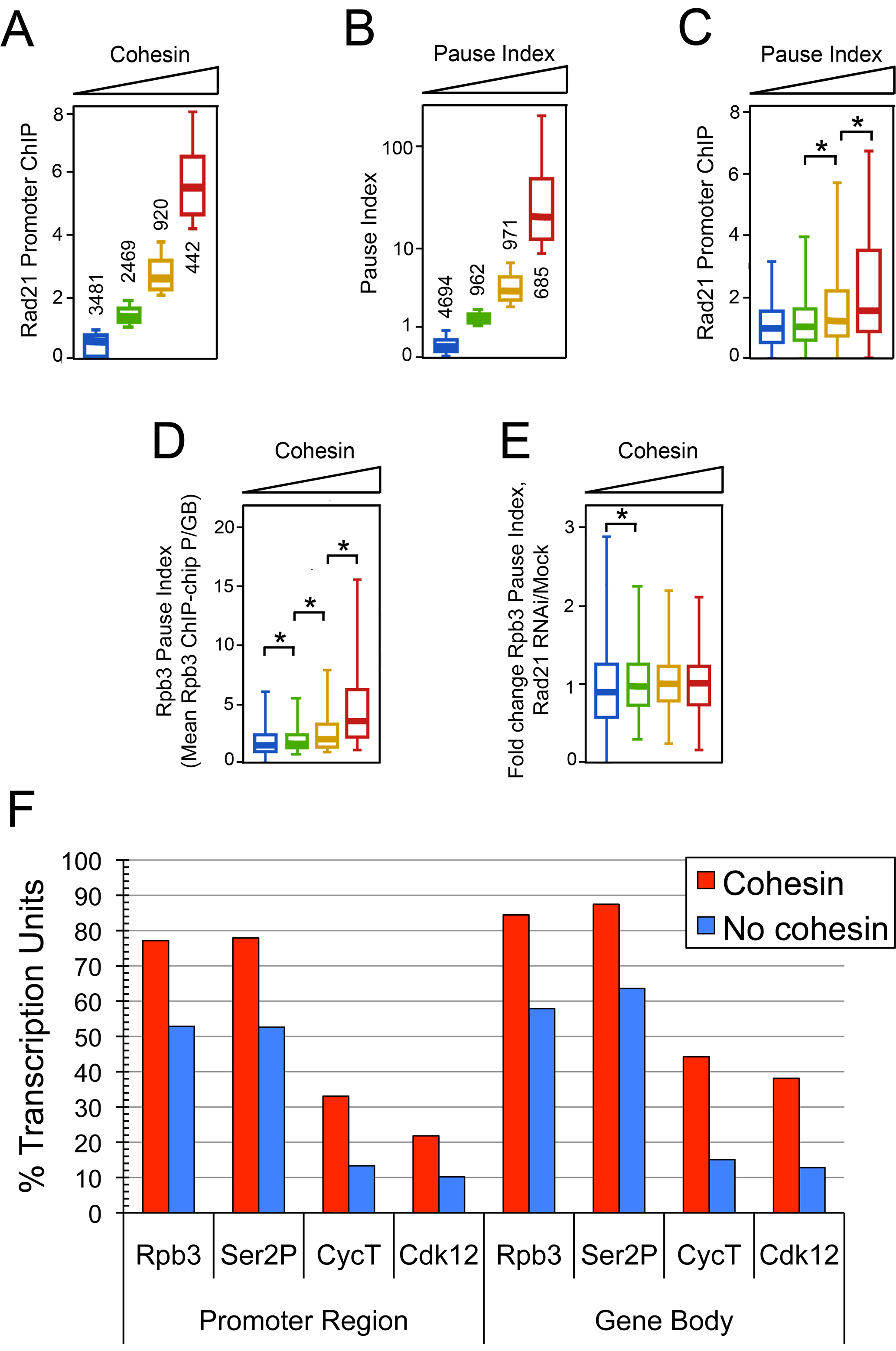

Supplement: Figure S1 — Cohesin binding genes have higher promoter-proximal pausing and Pol II occupancy. (A) Cohesin-binding groups of active genes used in Figure 1 based on levels of cohesin at the promoter. The numbers above or below each box plot indicate the number of genes in each group. (B) PRO-seq active genes distributed into groups based on pause index. (C) Cohesin (Rad21) occupancy of genes with increasing pausing. (D) Pause index of cohesin-binding groups calculated using ChIP-chip data [mean Rpb3 ChIP signal at promoter (P) divided by mean ChIP signal in gene body (GB)]. (E) Fold-change in pause index of cohesin-binding groups measured by Rpb3 ChIP upon Rad21 deletion. (F) Percent of transcription units with Rpb3, Ser2P Pol II, CycT (P-TEFb), and Cdk12 ChIP signal in the 200 bp promoter region surrounding the annotated transcription start site and the gene body at p≤10−3. The genes were divided in cohesin-binding and non-binding by Smc1 and Nipped-B occupancy [9] of the promoter region at p≤10−3. (TIF) [file pgen.1003382.s001.tif]

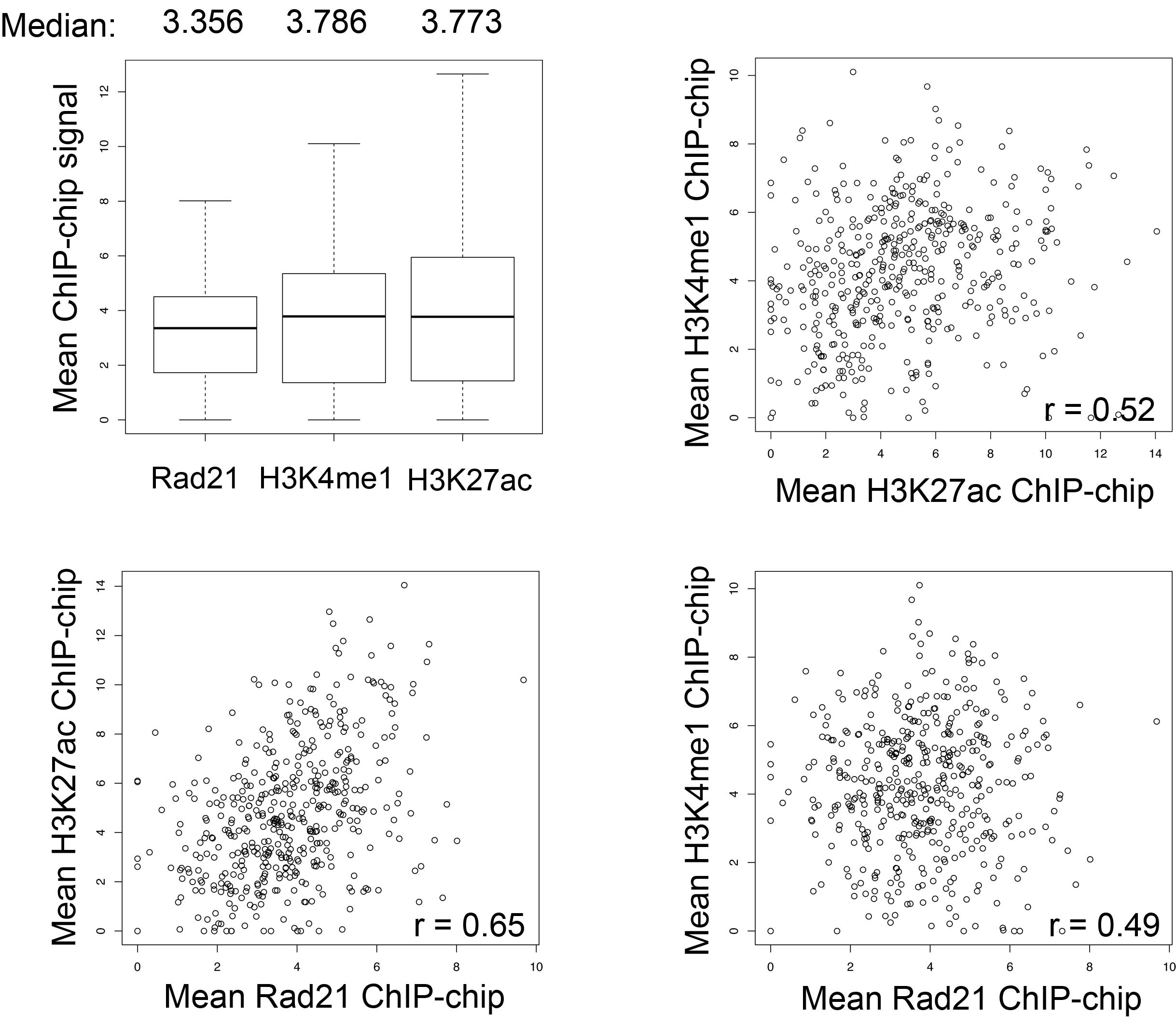

Supplement: Figure S2 — Cohesin binding correlates with the H3K27ac and H3K4me1 histone modifications at extragenic cis regulatory modules (CRMs). The box plots in the upper left panel show the distributions of the cohesin, H3K27ac and H3K4me1 ChIP signals at 557 extragenic CRMs (Table S3). The remaining panels plot the indicated ChIP signals against each other at each extragenic CRM and give the corresponding correlation coefficients. (TIF) [file pgen.1003382.s002.tif]

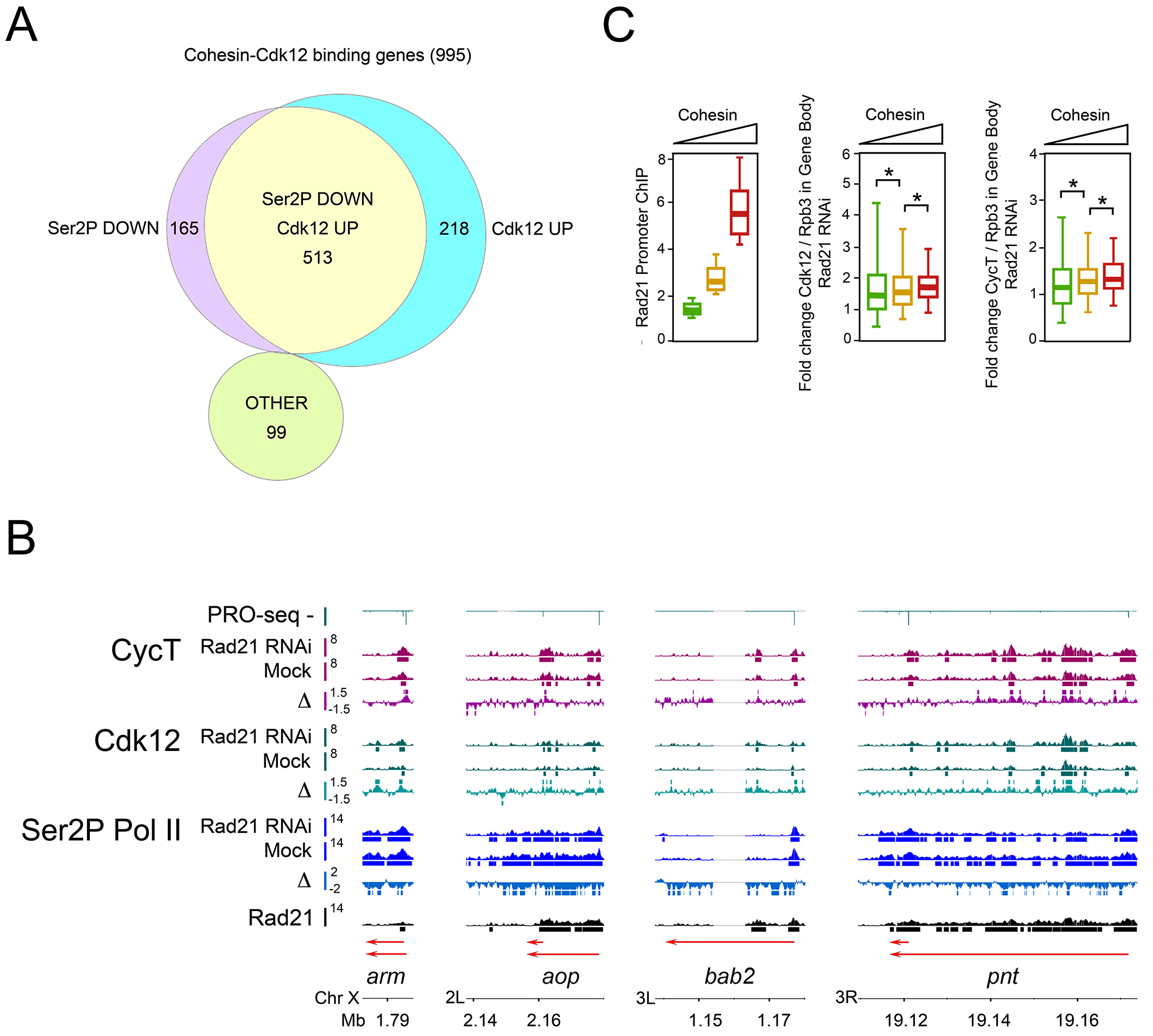

Supplement: Figure S3 — Cohesin depletion increases Cdk12 occupancy in gene bodies that have decreased phosphorylated Pol II. (A) Overlap of cohesin-Cdk12 binding genes with decreases in Ser2P Pol II in the gene body and increases in Cdk12. “Other” indicates no significant changes, an increase in Ser2P Pol II, or a decrease in Cdk12. (B) Examples of genes with Ser2P Pol II decreases in the gene body and an increase in Cdk12 and/or CycT. The top tracks show the PRO-seq reads in the control cells. The PRO-seq scale is 1000 for arm, bab2 and pnt, and 4000 for aop. The Δ tracks below the ChIP tracks show the difference in MAT score between the Rad21 depleted and control cells. The bars above and below the Δ tracks show where increases and decreases are ≥2 σ for regions ≥105 bp. (C) The left panel shows the Rad21 (cohesin) levels for the genes used in this analysis. They are three of the four groups shown in Figure S1A. The kinase ChIP signals in the group with the lowest cohesin binding were often too low to generate reliable fold-change ratios and kinase to Pol II ratios. The middle and right panels show the fold-change in the ratio of Cdk12 and CycT to Rpb3 in the gene body for each of the three cohesin-binding groups. (TIF) [file pgen.1003382.s003.tif]

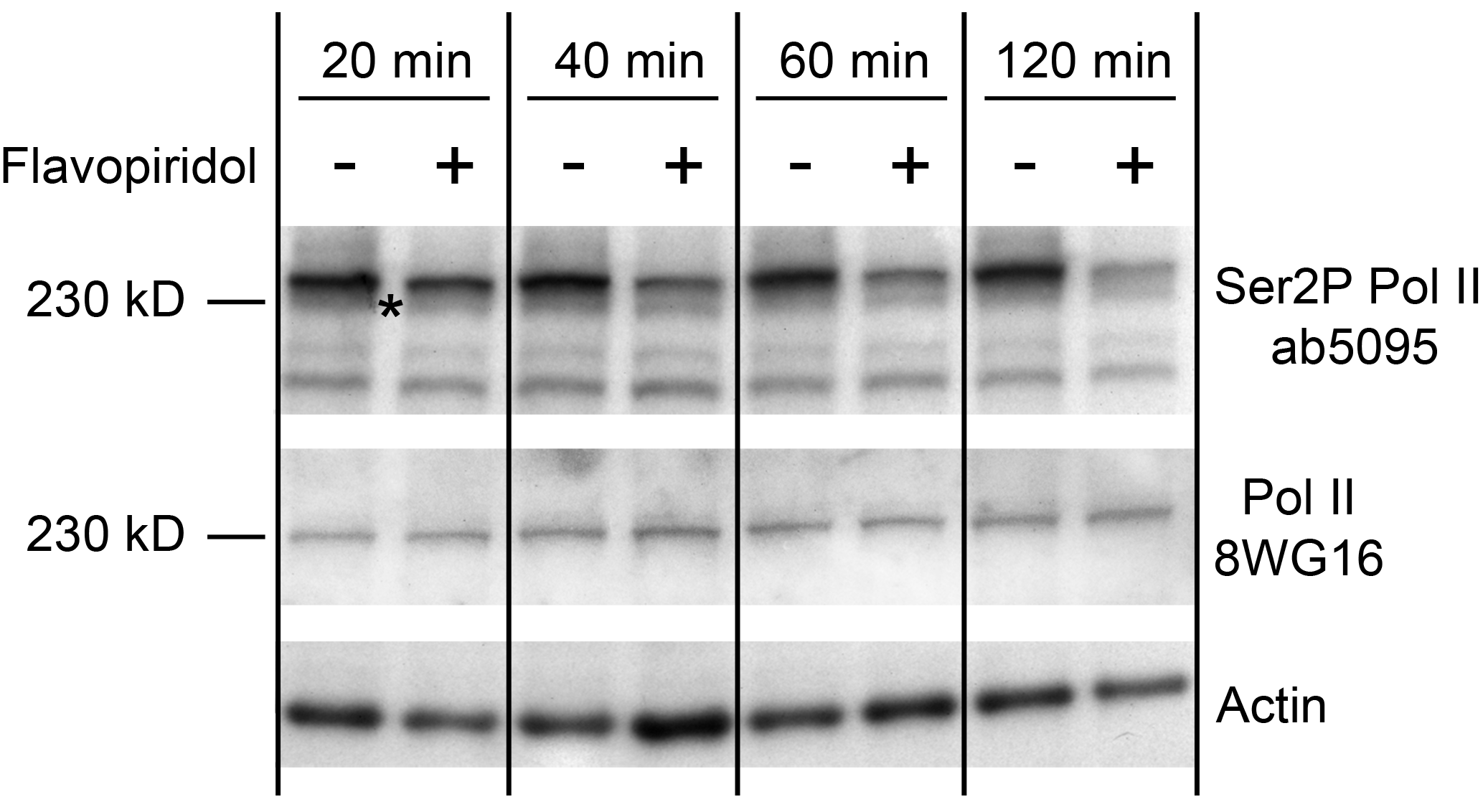

Supplement: Figure S4 — Validation of Ser2P Pol II antibody. The panels show a western blot of whole cell extracts of BG3 cells mock treated or treated with 1 µM flavopiridol, a P-TEFb inhibitor. The top panel shows the signal obtained with Ser2P Pol II antibody (Abcam ab5095) diluted 1∶1000. The middle panel is the same western reprobed with the 8WG16 antibody that recognizes primarily non-phosphorylated Rpb1, and the bottom panel shows the same blot probed with anti-actin as an internal standard. The asterisk (*) indicates a band that co-migrates with non-phosphorylated Rpb1, indicating that ab5095 antibody may slightly cross-react with non-phosphorylated Rpb1. (TIF) [file pgen.1003382.s004.tif]

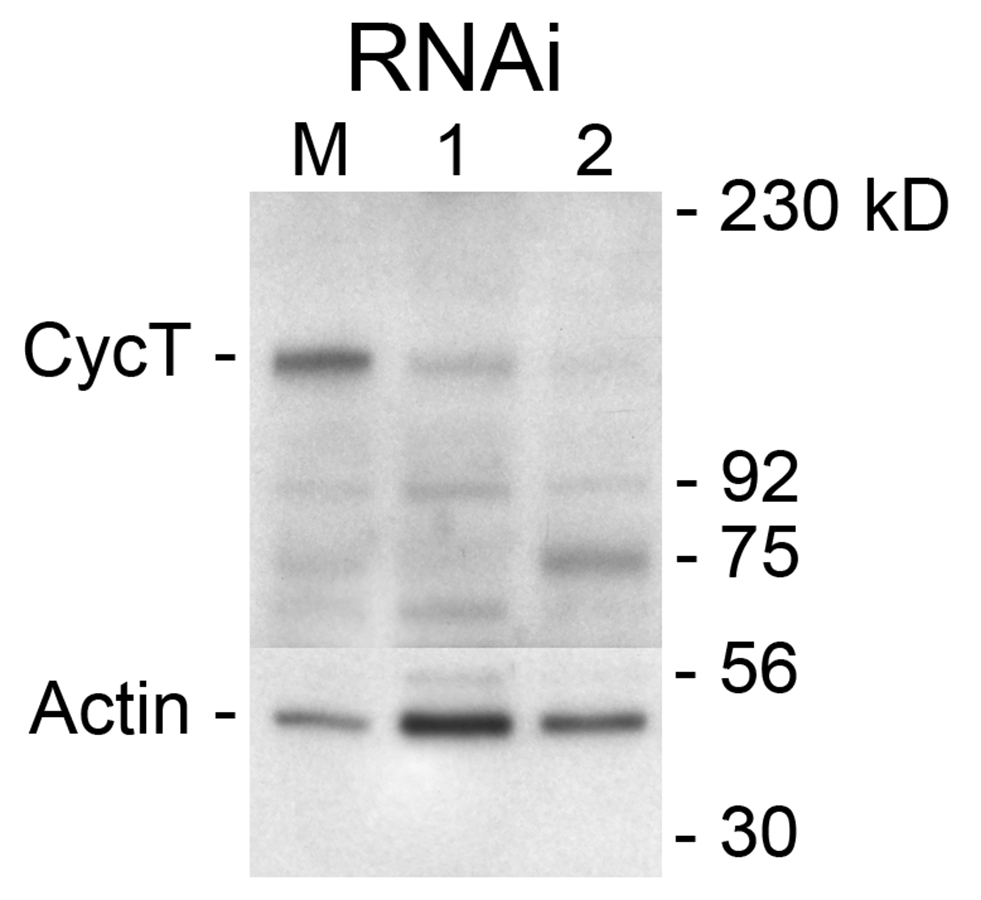

Supplement: Figure S5 — Validation of CycT antibody. BG3 cells were mock treated (M) or treated with one of two different dsRNAs (1 and 2) against with CycT for 3 days. Templates for synthesis of dsRNA were made by PCR from genomic DNA and dsRNA was prepared as previously described [13]. The PCR primers used to make template for dsRNA 1 were 5′-TAATACGACTCACTATAGGGAGACTCTTCCCAATGAGCCTCTG-3′ and 5′-TAATACGACTCACTATAGGGAGACATGGATGGTGGTACAGCAG-3′, and for dsRNA 2 5′-TAATACGACTCACTATAGGGAGACAAGCTAAATAGCCATCCGC-3′ and 5′-TAATACGACTCACTATAGGGAGAGGCGTGTGTTTCTCCTCAT-3′. Proteins were extracted from cells with buffer (10 microliters per ∼5×10^5^ cells) containing 40 mM Tris-HCl pH 7.4, 8 M urea, and 1% NP-40. After SDS-PAGE (∼2.5×10^5^ cells per lane) on a 4–20% gradient gel (Biorad TGX), proteins were electrotransferred to Immobilon-P membrane in buffer contain 100 mM CAPS pH 10.8 and 10% methanol. The western blot was probed with a 1∶1000 dilution of the CycT rabbit antiserum [45]. (TIF) [file pgen.1003382.s005.tif]
