# Supplementary material for: Genome-Wide Control of RNA Polymerase II Activity by Cohesin
Source: PLoS Genet. 2013 Mar 21;9(3):e1003382. doi: 10.1371/journal.pgen.1003382 (PMC3605059; doi:10.1371/journal.pgen.1003382)
Supplement: Table S1 — PRO-seq sequencing statistics. (DOCX) [file pgen.1003382.s006.docx]

**Table S1. PRO-seq sequencing statistics**

| Library | Total reads | Non-ribosomal | Mapped reads | Percentage |
| --- | --- | --- | --- | --- |
| Mock_1 | 26430533 | 18334276 | 14146725 | 54% |
| Mock_2 | 26629310 | 19047996 | 14597319 | 55% |
| Nipped-B_1 | 27994984 | 20784198 | 15936122 | 57% |
| Nipped-B_2 | 33526364 | 25613004 | 19819340 | 59% |
| Rad21_1 | 26639911 | 20049395 | 15592923 | 59% |
| Rad21_2 | 27996583 | 21242804 | 16523448 | 59% |
|  |  |  |  |  |
| Pearson correlation coefficients | Promoter proximal | Gene body |  |  |
| Mock | 0.97 | 0.98 |  |  |
| Nipped-B | 0.98 | 0.98 |  |  |
| Rad21 | 0.98 | 0.98 |  |  |
